# F189. PERSONALIZED MEDICINE: ESTIMATING THE IMPACT OF GENOTYPES ON ANTIPSYCHOTIC EFFICACY USING A QUANTITATIVE SYSTEMS PHARMACOLOGY APPROACH

**DOI:** 10.1093/schbul/sby017.720

**Published:** 2018-04-01

**Authors:** Hugo Geerts, Athan Spiros

**Affiliations:** In Silico Biosciences

## Abstract

**Background:**

CNS disorders are lagging behind other indications such as oncology in implementing genotype-dependent treatment algorithms for personalized medicine. This is due to the limited knowledge about the interaction of the relevant biology and the drug’s pharmacology

**Methods:**

We applied a mechanism-based computer model of a cortico-striatal-thalamocortical loop of the dorsal motor circuit that has been calibrated with clinical data on antipsychotic treatment in schizophrenia patients (Spiros, Roberts et al. 2017). The Quantitative Systems Pharmacology (QSP) model is based on the appropriate connections between basal ganglia regions and consists of 220 neurons (8 different cell types), 3500 synapses and implementations of 32 CNS active targets, based on their unique locations and coupling with intracellular pathways. COMTVal156Met, 5-HTTLPR rs 23351 s/L and D2DRTaq1A1 genotypes are implemented using human imaging data in non-medicated human volunteers

**Results:**

The dose-dependent antipsychotic effect for risperidone, aripiprazole and paliperidone is sensitive to the COMT genotype with the MM genotype having the greatest difference with the wild-type. Interestingly the 5-HTTLPR genotype interacts with the COMT genotype: this difference is positive for 5-HTTLPRss and negative for 5-HTTLPR LL. Olanzapine, quetiapine, clozapine and haloperidol are affected much less. The D2DRTaq1A allele interacts in a complex way with the COMT genotype with haloperidol, aripiprazole and risperidone and with the 5-HTLLPR genotype for haloperidol, aripiprazole, risperidone and paliperidone. These effects are anticipated to be detectable in clinical settings.

**Discussion:**

The QSP platform predicts strong and complex genotype-dependent interactions with aripiprazole, risperidone, haloperidol and paliperidone and to a much smaller degree with olanzapine, quetiapine and clozapine. These predictions could in principle be verified in clinical setting and could lead to rational personalized treatment guidance

